# Core–shell grain structures and ferroelectric properties of Na_0.5_K_0.5_NbO_3_–LiTaO_3_–BiScO_3_ piezoelectric ceramics

**DOI:** 10.1016/j.dib.2015.04.002

**Published:** 2015-04-20

**Authors:** Fangyuan Zhu, Michael B. Ward, Jing-Feng Li, Steven J. Milne

**Affiliations:** a State Key Laboratory of New Ceramics and Fine Processing, School of Materials Science and Engineering, Tsinghua University, Beijing 100084, China; b Institute for Materials Research, University of Leeds, Leeds LS2 9JT, UK

**Keywords:** Core–shell structure, Lead-free, NKN-based, Ferroelectric, TEM, Melting

## Abstract

Legislation arising from health and environmental concerns has intensified research into finding suitable alternatives to lead-based piezoceramics. Recently, solid solutions based on sodium potassium niobate (K,Na)NbO_3_ (KNN) have become one of the globally-important lead-free counterparts, due to their favourable dielectric and piezoelectric properties. This data article provides information on the ferroelectric properties and core–shell grain structures for the system, (1−*y*)[(1−*x*)Na_0.5_K_0.5_NbO_3_ – *x*LiTaO_3_] – *y*BiScO_3_ (*x*=0–0.1, *y*=0.02, abbreviated as KNN–*x*LT–2BS). We show elemental analysis with aid of TEM spot-EDX to identify three-type grain-types in the KNN–LT–BS ternary system. Melting behaviour has been assessed using a tube furnace with build-in camera. Details for the ferroelectric properties and core–shell chemical segregation are illustrated.

**Specifications table**

**Table:** 

Subject area	*Materials science*
More specific subject area	*Piezoelectric ceramics*
Type of data	*image (STEM-scanning transmission electron microscope images; photographic images of pellets during high-temperature processing), graph ( ferroelectric behaviour and corresponding spot EDX results)*
How data was acquired	*By transmission electron microscopy (FEI Tecnai F20, EDX from the Oxford Instruments plc, Abingdon, UK) and Precision LC Ferroelectric Tester (Radiant Technologies, Inc.)*
Data format	*Raw, analysed data*
Experimental factors	*TEM specimens were FIBed (focus ion beamed) for ultra-thin samples; ferroelectric analyser was utilised for ceramic discs.*
Experimental features	1. *TEM spot-EDX specimens were prepared by focused ion beam milling and in- situ lift out.* 2. *Polarisations vs. electric field measurement were obtained by using a precision LC ferroelectric tester manufactured by Radiant Technologies.*
Data source location	*IMR-SPEME, University of Leeds, Leeds, Yorkshire, UKSMSE, Tsinghua University, Beijing, China*
Data accessibility	*The data is with this article.*

**Value of the data**

• Ferroelectric response under external electric field of KNN–*x*LT–2BS ceramics were measured, for compositions *x*=2, 5, 6 mol%.
• High-angle annular dark-filed imaging (HAADF) with STEM are used to probe chemical inhomogeneity within grains of KNN–45T–2BS and KNN–6LT–2BS ceramic samples.
• Spot-EDX data for nano-regions within the KNN–6LT–2BS core–shell grains.
• STEM-HAADF shows influence of excess alkali metal carbonates in the starting powder mixture on chemical segregation/core–shell structures.
• Melting surveillance pictures were recorded as a function of furnace temperature for KNN–*x*LT–2BS compositions *x*=0, 2, 5, 10 mol%.

## 1 Data, experimental design, materials and methods

**Fig. 1:**
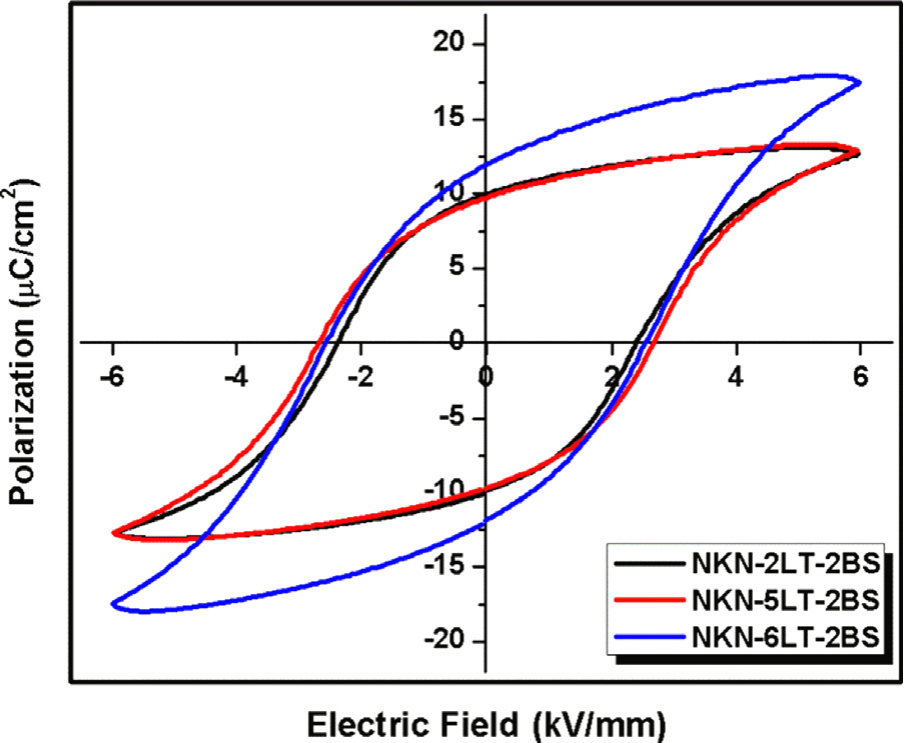
Polarisation hysteresis loops under 6 kV/mm external field for NKN–*x*LT–2BS when *x*=2, 5 and 6 mol% at room temperature.

We present ferroelectric and dielectric properties for core–shell grain compositions within KNN–*x*LT–2BS solid solutions. Fig. 1 shows the coercive fields (*E*_*c*_) for various LT concentrations for samples with and without core–shell grain microstructures. The 2LT sample is chemically uniform without core–shell grain structures [1] and the *E*_*c*_ is ~2.35 kV/mm. Identical core–shell grain structure appeared in samples that had increased the LT to 6 mol%, while the *E*_*c*_ is ~2.50 kV/mm.

Spot-EDX analyses using traditional TEM were performed on specimens fabricated by FIB-SEM (for which details and processing conditions have been described previously [1,3–5]). The TEM beam spot size was ~5 nm diameter, and the actual volume analysed was approximately 2000 nm^3^. This is assuming a cylinder of length 100 nm (corresponding to the sample thickness) is analysed and neglecting beam spreading in sample. This is equal to around 30 unit cells. Several spot-EDX analysis were taken for KNN–*x*LT–2BS, *x*=4, 5 and 6 mol%; chemical formula were calculated from the EDX data to better understand the chemical variations within the samples. This distinctive method was utilized to separate the chemical compositions within segregated parts of grains, details can be found in [5].

Three microstructure/dielectric property classifications were adopted for KNN–*x*LT–2BS solid solutions, which are listed as:

*Type I*: for *x*=0–2 mol% LT compositions; single, sharp dielectric Curie peak (~370 °C); single phase by XRD; large grain size (5–10 μm); chemically uniform by TEM-EDX.
*Type II*: for *x*=3–4 mol% LT compositions; broad, single dielectric peak (~350 °C); single phase by XRD; large grain size; no obvious chemical segregation.
*Type III*: for *x*=5–10 mol.% LT compositions; twin, broad dielectric peak(s) (~370 °C and~470 °C); broad XRD peaks; small grain size (~0.5 μm); chemical segregation (core–shell structure) identified by TEM-EDX [4,5].

**Fig. 2:**
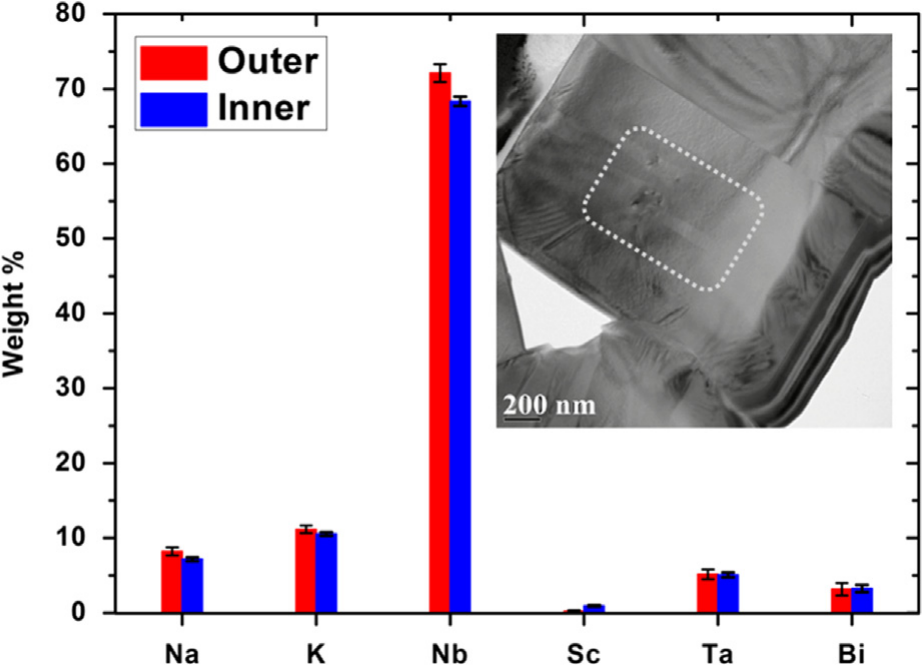
NKN–4LT–2BS specimen: averaged EDX spots analysis across the grain presented as histogram in main feature. Inner region is defined by a dashed line (inset figure) but results indicate an absence of core–shell chemical segregation with the grain.

**Fig. 3:**
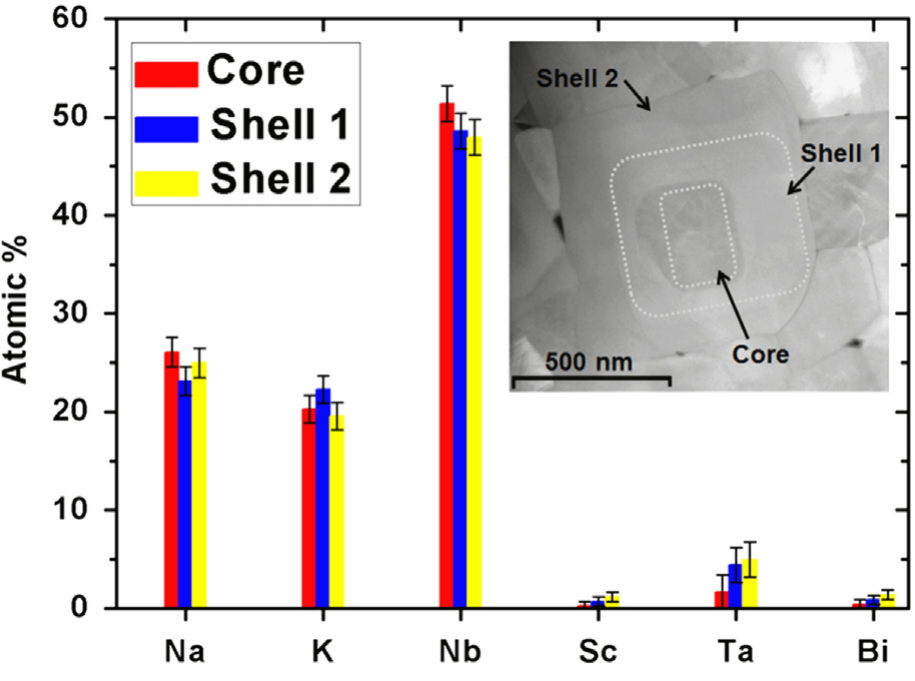
NKN–6LT–2BS specimen: integrated EDX spectral analysis from different regions of a grain with three-tier contrast in. (Inset TEM highlights Core/Shell 1/Shell 2).

Fig. 2 presents the *Type II* NKN–4LT–2BS specimen which has no measurable chemical concentration gradient across component grains. Increasing the LT content to KNN–6LT–2BS, produces core–shell grains with a novel three-tier metastable grain structure, but the proportion of core–shell grains in the microstructure is lower than for the 5LT sample. Spot-EDX confirmed that the outer shell (labelled as Shell 2 in Fig. 3) of three-tier structures is slightly rich in Sc, Ta, Bi. The EDX data for the middle shell (shown as Shell 1 in Fig. 3) was similar to the outer shell, whilst the core part was deficient in Ta.

**Fig. 4:**
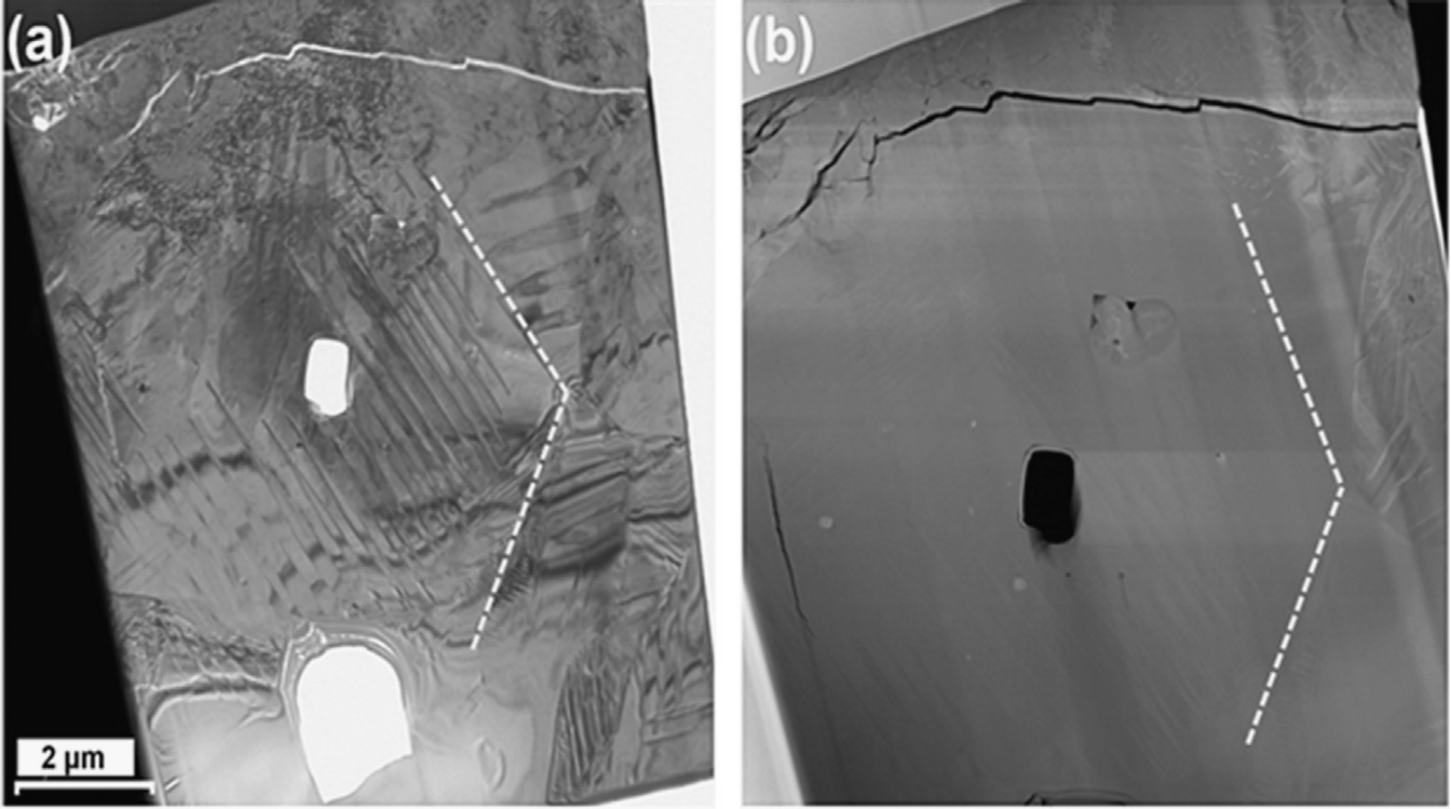
*Excess* NKN–6LT–2BS specimen: (a) bright field TEM observation; and (b) HAADF image for the same grain (dashed line indicates the grain boundary).

An excess of 3 mol% alkali carbonates was mixed to KNN–6LT–2BS starting powders, which named as *Excess* KNN–6LT–2BS. The microstructure of this *Excess* sample has a relative large grain size ~4–5 µm and no chemical concentration by STEM-HAADF and spot-EDX exanimations, Fig. 4.

**Fig. 5:**
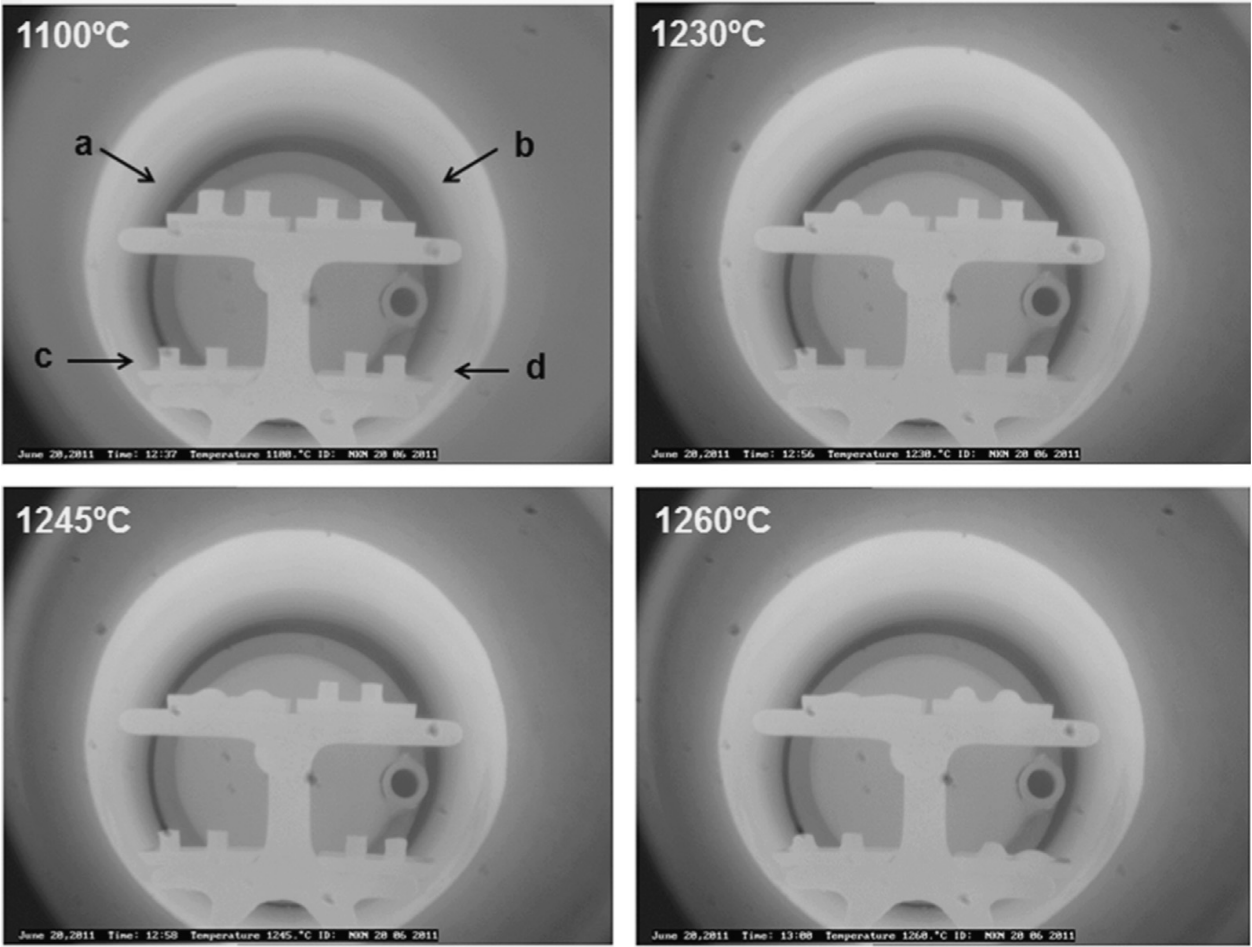
Melting temperature test for NKN–*x*LT–2BS powder compacts. The images of the cylindrical compacts at different temperatures on a heating cycle were captured by a built-in camera in a home-made tube furnace: (a) NKN (Na_0.5_K_0.5_NbO_3_), (b) NKN–2LT–2BS, (c) NKN–5LT–2BS and (d) NKN–10LT–2BS. Starting pellets were 0.5 cm in diameter and 1 cm in height. Two pellets of each composition were prepared. (Notice: NKN–5LT–2BS (c) shows least distortion at high temperature indicating higher melting temperature.)

Fig. 5 shows images of pellets as a function of temperature which can be seen through a home-built tube furnace with a viewing window. This permitted visible evidence of deformation, shrinkage and melting process. All three-types of sample microstructures were tested from room temperature to 1350 °C as clarified in Fig. 5; images were recorded every 5 °C. All the compacts shrank at 1100 °C, which was the normal sintering temperature for this study. KNN–5LT–2BS powder compacts (no excess alkali carbonate, core–shell structure grains sample) retained their shape to a higher.
